# Parkinsonism with a Hint of Huntington’s from 29 CAG Repeats in HTT

**DOI:** 10.3390/brainsci9100245

**Published:** 2019-09-22

**Authors:** Sipilä JOT

**Affiliations:** 1 Department of Neurology, Siun Sote North Karelia Central Hospital, 80120 Joensuu, Finland; jussi.sipila@utu.fi; 2 Division of Clinical Neurosciences, Turku University Hospital, 20521 Turku, Finland; 3 Department of Neurology, University of Turku, 20520 Turku, Finland

**Keywords:** clinical neurology, differential diagnosis, genetics, neurodegenerative disease, parkinsonism, movement disorders, neurophysiology

## Abstract

Huntington’s disease is caused by at least 36 cytosine-adenine-guanine (CAG) repeats in an HTT gene allele, but repeat tracts in the intermediate range (27–35 repeats) also display a subtle phenotype. This patient had a slightly elongated CAG repeat tract (29 repeats), a prominent family history of Parkinson’s disease (PD), and a clinical phenotype mostly consistent with PD, but early dystonia and poor levodopa response. Neurophysiological test results were more consistent with Huntington’s disease (HD) than PD. It is suggested that the intermediate allele modulated the clinical phenotype of PD in this patient.

## 1. Introduction

Huntington’s disease (HD) is caused by a cytosine-adenine-guanine (CAG) repeat tract of at least 36 repeats in the HTT gene while intermediate alleles (IAs, 27–35 CAG repeats) are associated with a subtle, or sometimes even an overt phenotype [1,2,3,4]. The typical movement disorder in HD is characterized by both hyper- and hypokinesia with hyperkinesia more prominent in the early stages. However, in the rare Westphal variant, most often found in patients with disease onset before adulthood, hypokinesia predominates from the onset. Interestingly, HD has also been reported to manifest as a levodopa-responsive multiple system atrophy (MSA) [5]. Moreover, IAs have been reported to even cause manifest HD in some cases but this is being debated [6,7,8,9,10]. The reported prevalence of IAs on the population level in Caucasians is 6%, but this is dependent on the chromosome 4 haplotype distribution in the population and is therefore probably much lower in Finland where the haplotype distribution differs from other Western European populations [11,12,13]. Here is presented a patient with a phenotype largely consistent with akinetic-rigid Parkinson’s disease but with peculiar features suggesting that the intermediate HTT allele (29 CAG repeats) may have modulated the phenotype.

## 2. Case Report

The patient presented at the age of 71 years with gait difficulties that had developed during the preceding year and characterized by slowing of ambulation and involuntary bending of the toes when walking. He had also developed hypokinesia in all activities, deterioration of handwriting, constipation and a dry cough. On examination, hypokinesia and hypomimia were present along with a forward stooping posture, short and shuffling gait, impaired balance control and clumsiness of fine motor control. The signs were more obvious on the left side and he also leaned left when walking. According to the patient, his sister had been diagnosed with PD at the age of 50 years and died at the age of 64 years. His maternal uncle had also developed PD at the age of 60–70 years. The patient records for these relatives are not available.

**Figure 1:**
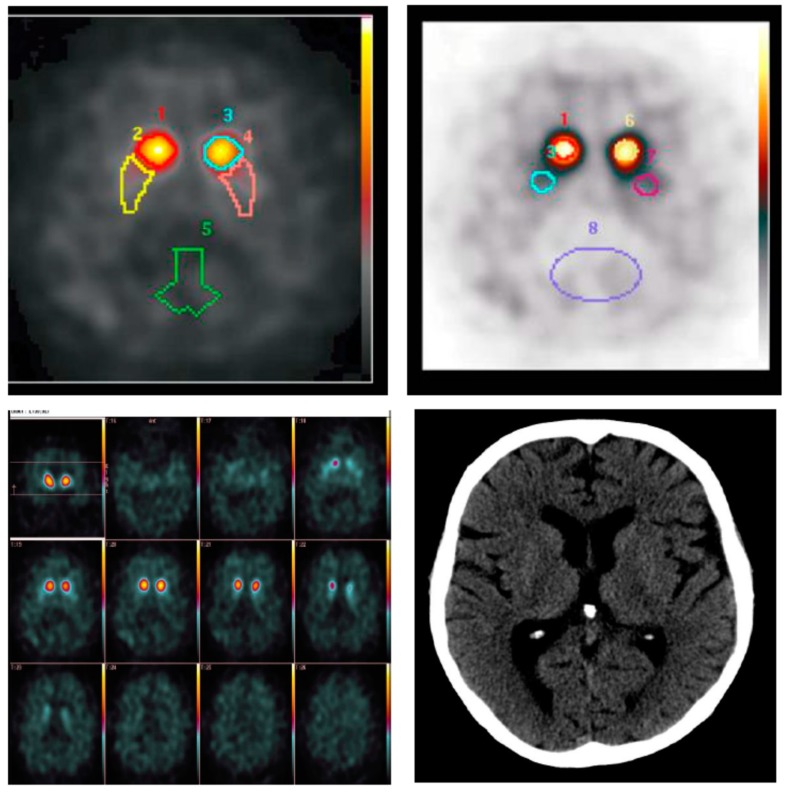
Beta-CIT-Spect and non-contrast computed tomography imaging excerpts from the time of diagnosis.

Beta-CIT Spect revealed symmetric and severe depletion of dopamine transporter proteins in the putamina and slightly less severe, diffuse depletion in the caudate nuclei (Figure 1). There were minor changes in thyroid function testing that were normalized after the initiation of thyroxine replacement therapy. Creatine kinase levels were slightly elevated (up to 500) and an extensive muscle disease work-up (including e.g., MRI imaging and genetic panel testing) was performed leading only to a diagnosis of axial and limb muscle myopathy and monoallelic mutations associated with recessively inherited diseases in AGL and SBF1 genes which were apparently incidental findings. Polysomnography revealed a severe sleep apnoea (oxygen desaturation index 37.5 /h) with desaturations down to 45% during rapid eye-movement (REM)-sleep. REM behavioral sleep disorder was not observed. Several tests, including head non-contrast computed tomography (Figure 1), bronchoscopy and colonoscopy, lung function tests, and many routine blood tests, provided normal results.

The patient was treated with levodopa (up to 600 mg per day with entacapone), ropinirole and amantadine along with physical therapy. These proved of little benefit, although discontinuation (because of no apparent benefit) of amantadine at four years from symptom onset led to profound rigidity and difficulties in the initiation of walking which both improved when amantadine was continued. Amitriptyline, gabapentin and tizanidine were prescribed for pain with also a modest effect at best. Botulinum toxin injections were tried for the feet to no effect.

Five years after symptom onset the patient was alert and oriented. He had camptocormia but no Pisa sign. His speech was dysarthric and therefore difficult to understand at times. No cognitive decline was evident and CERAD had been normal a year earlier. There were no observations of psychiatric symptoms or hallucinations. There was only slight postural and action tremor evident in the upper limbs with no resting tremor. There were no dyskinetic or choreatic movements despite dopaminergic medication. Slight rigidity was observed, mostly in the upper limbs. Bradykinesia was evident, the gait was shuffling. Postural reflexes were impaired, but he was able to walk unassisted in the examination room. He used a walker at home and an electric moped when moving outside. Saccade initiation was slightly slow, and saccades were slightly hypometric, but the eye movement range was full. He was able to keep his tongue protruded for 10 s without problems. There were only mild autonomic symptoms. Of clinical rating scales, Unified Parkinson’s Disease Rating Scale (UPDRS) III gave 40 points, Unified Huntington’s Disease Rating Scale (UHDRS) III 20 points, Unified Multiple System Atrophy Rating Scale (UMSARS) II 19 points and UMSARS III 17 points.

Because of the poor levodopa response (although very high doses were not tried) and previous reports of atypical manifestations of HD, HTT was analysed and the test showed alleles of 29 and 17 CAG repeats. To further differentiate between HD and PD, the blink reflex was tested with a result showing a swift habituation with normal R1 and R2 latencies.

A tendency to fall intermittently had developed seven years from symptom onset. At this time the patient also developed delusions and anxiety which were somewhat relieved with the discontinuation of ropinirole. CERAD was performed again with pathologic results only in Mini Mental State Examination (23/30 including full points in orientation and 4/5 points in serial-7 s) and constructional praxis recall (33%, while word recall was 75%). Two months after this he fell at home, sustaining a femoral fracture which was treated surgically. Recovery was slow and the patient died of pneumonia five weeks after the operation. No autopsy was performed. The family members were given genetic counselling, but none of them have opted for predictive testing.

## 3. Discussion

This patient’s phenotype was mainly consistent with Parkinson’s disease. Considering the MDS clinical diagnostic criteria for Parkinson’s disease [14], the patient manifested bradykinesia and asymmetric rigidity, two of the cardinal features of PD. There were also no absolute exclusion criteria or red flags present. Additionally, the signs and symptoms were better captured by UPRDS than UHDRS or UMSARS. On the other hand, he fulfilled no supportive criteria either and therefore a diagnosis of clinically established PD cannot be made. Considering that HTT IAs are known to exert discernible effects on physiology [1,2,3], it appears plausible that this patient’s IA affected the phenotype. Consistent with this, this patient’s blink reflex results habituated swiftly similarly to patients with HD whereas in PD delayed or no habituation has been reported [15,16,17]. Moreover, although the clinical phenotype in this patient was mostly consistent with PD, there was early dystonia when still untreated which is very uncommon in PD and, according to Tolosa and Compta, should raise the suspicion of other entities [18].

Considering the rarity of HD in Finland and the population genetic factors underlying this [12,13], it is unlikely that the association presented here was a mere coincidence. Indeed, the only study that has studied the matter so far in Finns reported an IA prevalence of 0.9% [11]. Furthermore, somatic mosaicism is well documented in HD with the repeat lengths longest in the brain regions that are the worst affected by the disease process [19,20,21]. Therefore, the IA may well exert more effects than could be deduced from the mere blood test analysis. Interestingly, a recent Spanish study reported an IA prevalence of 2.9% among healthy controls and 3.5% among patients with PD whereas the frequency was 5.3% among patients with frontotemporal lobar degeneration and 6.0% among patients with Alzheimer’s disease (AD) and the difference was statistically significant only between healthy controls and AD patients [22]. They also reported no atypical symptoms or clinical features distinctive of HD among IA carriers, but the analysis appears to primarily have been aimed at finding signs of HD among the carriers whereas the patient reported here was analysed in more depth. All in all, considering the early dystonia, the poor levodopa response and neurophysiology results suggestive of HD rather than PD as well as the clinical criteria, it appears that the HTT IA modulated the parkinsonism phenotype of this patient.
